# Supplementary material for: Dosimetric comparison of Monte Carlo, Acuros XB, and anisotropic analytical algorithm for lung cancer plans on halcyon accelerators
Source: PLoS One. 2025 Oct 17;20(10):e0334329. doi: 10.1371/journal.pone.0334329 (PMC12533868; doi:10.1371/journal.pone.0334329)
Supplement: Attachment — Submitted filename: Response_to_reviewers.pdf [file pone.0334329.s005.pdf]

Response_to_reviewers_haMC

Reviewer #1:

Title: Dosimetric comparison of VMAT lung cancer plans on Halcyon Accelerators using Monte Carlo, Acuros XB, and AAA algorithms

General comments:

This manuscript aims to compare dose distributions calculated by using the RayStation based on Monte Carlo (MC), Anisotropic Analytical Algorithm (AAA), and Acuros XB (AXB) to ensure dose calculation accuracy across different platforms. They also wanted to report dosimetric impact of choosing calculation algorithm on patient dose expectation. The TPS should be fast as possible while it ensures acceptable accuracy compatible to the MC, which has been regarded as a golden standard but requires large amount of time to calculate patient dose distribution. It is essential to validate the accuracy of TPS by comparing the MC simulation. Since many algorithms have been used in the medical institutions, in addition, guarantee of accurate dose calculation across different algorithms is necessary. However, there have been studies on comparing the MC, AAA, and AXB for several years even the beam or tumor site is different (Zaman, et al., 2019, Sarin et al, 2022, Tsuruta, et al., 2014, Han et al., 2011, Seniwal et al., 2020). Authors should suggest their unique findings different from previous studies, but I'm not sure with the current manuscript. I hope they provide them in the next revision of the manuscript or it may be better to submit the manuscript as a technical note.

Response to General Comments:

Thank you very much for your constructive feedback. We fully understand your concern regarding the novelty of our work and have carefully revised the manuscript to clarify how our study differs from previous publications (Zaman 2019, Sarin 2022, Tsuruta 2014, Han 2011, Seniwal 2020) and to highlight its unique contributions:

1. **Cross-TPS validation specifically for Halcyon accelerators**
   Previous studies mainly focused on C-arm linacs such as TrueBeam. To the best of our knowledge, no prior work has systematically investigated the dosimetric consistency between RayStation and Eclipse for Halcyon, a closed-system platform where clinical workflows often require MC-based planning in RayStation followed by dose recalculation using AXB or AAA in Eclipse for verification in ARIA. Our study is the first to directly address this practical need, providing robust evidence for cross-TPS dose consistency in Halcyon workflows.
2. **Larger cohort and higher target complexity**
   Most prior studies were limited to small samples, SBRT cases, or phantom-based evaluations (e.g., Tsuruta 2014 involved only 26 SBRT patients). In contrast, our study included **63 conventionally fractionated lung cancer patients** with larger and more anatomically complex targets, thereby better reflecting real-world clinical scenarios and enhancing the generalizability of our findings.
3. **Direct comparison between RayStation MC and Eclipse AXB/AAA**
   While earlier works focused on AAA vs. AXB or AXB vs. MC within a single TPS, our study uniquely examines the differences between RayStation MC and Eclipse AXB/AAA, mirroring actual clinical workflows. This is particularly relevant for institutions that are currently using

or planning to use RayStation for Halcyon treatment planning, as our findings provide clear guidance for safe and efficient cross-platform implementation.

4. **Clinical interpretation of algorithmic differences**
Beyond statistical comparisons, we analyzed the clinical relevance of the observed differences. Although some differences were statistically significant, the majority of them were <1% for target coverage, and OAR dose differences remained within clinically acceptable limits, thus supporting the practical interchangeability of these algorithms.
5. **Scope beyond a technical note**
Our study integrates phantom validation, a 63-patient clinical cohort, rigorous statistical analysis (including Bonferroni correction), and comprehensive dosimetric evaluation. Given the study scale and depth, we believe it is more appropriately presented as a full research article rather than a technical note.

We have revised the **Introduction** and **Discussion** sections of the manuscript to explicitly emphasize these points and to better delineate the incremental value of our study in comparison to prior literature.

Specific comments:

Comment 1: Avoid using abbreviations in the Title.
Response 1: We agree with the reviewer's suggestion. We have revised the title to remove abbreviations.

Comment 2: Use of RayStation must accompany with using Eclipse to apply the plans to Halcyon machine. Then, is there any advantages of using both the TPSs rather than solely using the Eclipse if the recalculation with the Eclipse must be carried out?

Response 2:

1. We appreciate the reviewer's question and have clarified this point in the revised manuscript. Although Eclipse is required for final dose recalculation and plan transfer to the Halcyon machine, our institution has historically used RayStation (previously Pinnacle) for most of our linear accelerators, including TrueBeam, Trilogy, Infinity, and Synergy. Radiation oncologists are accustomed to contouring and plan evaluation within RayStation, and maintaining the same TPS for both Halcyon and other linacs ensures workflow consistency, facilitates inter-machine plan transfer, and minimizes the learning curve for physicians and physicists.

In addition, RayStation offers unique advantages over Eclipse, including:

- **Advanced optimization features:** RayStation supports direct machine parameter optimization (DMPO), which enables efficient generation of high-quality plans.
- **GPU-accelerated Monte Carlo calculation:** The GPU-based MC algorithm in RayStation significantly reduces computation time while maintaining high accuracy, thereby improving planning efficiency.
- **Integrated workflow:** Using RayStation as the primary planning platform allows seamless plan evaluation across different machines, while Eclipse is used solely for final recalculation and delivery compatibility with Halcyon.

We have added this explanation to the **Discussion section** to clarify the institutional rationale and the benefits of this dual-TPS workflow.

Comment 3:

Even though the RayStation provides MC based calculation, I'm not sure it is compatible with the full-MC simulation as it seems to have adjusted algorithm to make the calculation faster. There may be assumptions to increase calculation speed.

According to my knowledge, the full-MC has been considered as a gold standard. I wonder that the RayStation can be considered same as the full-MC simulations using the codes such as EGS, Geant4, TOPAS, etc. I think it would be better to include Full-MC simulation result.

Response 3:

Thank you for this insightful comment. We fully agree that research-grade full Monte Carlo (MC) simulations (e.g., EGSnrc, Geant4, TOPAS) are regarded as the reference standard for dose calculation accuracy. Although we did not include new full-MC simulations in this study due to their substantial computational cost, several published studies have validated the RayStation MC algorithm against EGSnrc-based codes and experimental measurements.

Richmond et al. (2021) {10.1016/j.ejmp.2021.02.002} demonstrated that the RayStation MC photon dose engine, which is physically inspired by EGSnrc, achieved $>$95% gamma pass rates (2%/2 mm) compared with DOSXYZnrc in heterogeneous geometries. Manco et al. (2023) {10.1016/j.ejmp.2023.102588} further confirmed that RayStation MC maintains a dosimetric accuracy within 2–3% in both homogeneous and heterogeneous conditions for multiple linacs, meeting the clinical tolerance criteria recommended by IAEA TRS-430. These studies collectively support the equivalence of RayStation MC to full-MC simulations for clinical dose calculation purposes.

We have added this clarification and the corresponding references to the revised manuscript.

Comment 4:
It would be better to provide the required calculation time for obtaining dose distribution with the RayStation since it was considered as the MC calculation and authors explained statistical uncertainty and size of the calculation grid.

Response 4:

Thank you for this helpful suggestion. We agree that including the calculation time and technical parameters would improve the clinical context of our study. In our study, the final dose calculation for a typical VMAT plan for conventionally fractionated lung cancer patients using the RayStation Monte Carlo algorithm was performed with a dose grid size of **2.5 mm** and a statistical uncertainty of **0.2% per plan**, requiring approximately **20–30 seconds** on a GPU workstation equipped with an **NVIDIA RTX A6000 (24 GB)**. These results confirm the high computational efficiency of RayStation MC and support its practical integration into routine clinical workflows.

We have added this information to the Methods and Discussion sections of the revised manuscript.

Comment 5:
The authors defined the ROIs in the water phantom with different radii, 3 and 5 cm. Is the reason for this to consider both high and low dose region?

The authors should also provide statistical constraints in the MC calculation in the water phantom study as did they in patient case study.

Response 5:
Thank you for these comments.

- Regarding the ROI sizes in the water phantom study:
  Yes, the spherical ROIs with radii of 3 cm and 5 cm were selected to evaluate both high-dose and low-dose regions in the phantom. The 3 cm ROI was used to assess the high-dose uniform region within the field, while the 5 cm ROI extended toward the periphery of the field and better represented the dose gradient and lower-dose regions. We have added this clarification to the Methods section.
- Regarding the MC statistical constraints in the water phantom study:
  We used the same statistical uncertainty as for the patient plans (0.2% per plan), but a finer dose grid size of 2 mm was used in the phantom study to better resolve the high-gradient regions. This information has been added to the Methods section of the revised manuscript.

Comment 6:
The authors categorized their cohort according to tumor stage. Is there any reason for this? This manuscript is not related to treatment outcomes or other biological effects.

Response 6:
Thank you for this comment. The tumor stage information was included only to provide a clear description of the patient cohort, as reporting baseline characteristics such as stage is a common requirement in radiotherapy studies and is often requested by reviewers to ensure adequate cohort transparency.

Comment 7:
Authors stated they used three algorithms: haMC, haAXB, and haAAA. However, I guess these are labels of data, not the name of the algorithm. I think they should be changed to MC, AXB, and AAA.
Response 7:
Thank you for pointing this out. We agree that the correct algorithm names are Monte Carlo (MC), Acuros XB (AXB), and Anisotropic Analytical Algorithm (AAA). In our original manuscript, the labels "haMC," "haAXB," and "haAAA" were used not to rename the algorithms but to indicate that the corresponding dose calculations were performed for Halcyon plans.

To avoid confusion, we have:

Clarified in the Methods section that the prefix "ha" is only a dataset label specifying that the plans were generated for the Halcyon accelerator, while the algorithms themselves remain MC, AXB, and AAA.

Updated the figure legends to explicitly state that "ha" indicates Halcyon-specific datasets, whereas the algorithm names remain unchanged.

This revision should eliminate ambiguity while preserving the distinction between algorithm names and dataset labels.

Comment 8:

According to the authors, did the AXB also produce dose deviations in boundary regions since the gamma pass rate of AXB&AAA was higher than that of MC&AXB? The gamma pass rate was remarkably increased and this needs to be clarified.
Response 8:
Thank you for this comment. The slightly higher global gamma pass rate between AXB and AAA

(100%) compared to MC and AXB (99.57%) does not indicate that AXB introduces larger deviations in boundary regions. Global gamma evaluation is less sensitive to small discrepancies in high-gradient regions because it normalizes the dose difference to the global maximum dose.

In contrast, the local gamma analysis, which is more sensitive to boundary dose variations, demonstrated excellent agreement between MC and AXB (99.03%) and a markedly lower pass rate between MC and AAA (87.87%). This confirms that MC and AXB are in closer agreement in heterogeneous boundary regions, whereas AAA tends to overestimate dose in these areas.

Moreover, both the global and local gamma pass rates between AXB and AAA were very high (100% and 95.07%, respectively). This may reflect the fact that AXB and AAA share similar underlying dose-calculation approximations, as both are implemented within the Eclipse system, which leads to greater agreement between them even though AAA is less accurate in heterogeneous boundary regions.

We have revised the manuscript to clarify these points.

Comment 9:
It would be better to provide gamma analysis for the patient case study similar to the water phantom study.

Response 9:
Thank you for this comment. In clinical practice, gamma analysis is primarily performed in phantom-based patient-specific quality assurance (PSQA) according to AAPM TG-218 recommendations (e.g., ArcCHECK or Delta4), rather than on patient CT dose distributions. This is because gamma analysis on CT images is highly sensitive to anatomical heterogeneities and does not necessarily reflect the clinically relevant dose–volume differences for targets and OARs, which are the focus of treatment evaluation.

For this reason, our study evaluated dose consistency between algorithms primarily using DVH-based metrics for target volumes and OARs, which are more clinically meaningful.

Nevertheless, to address the reviewer's suggestion, we performed an additional exploratory gamma analysis on two representative patient cases using 10% dose threshold, 3%/3 mm criteria. For patient 15 (PTV volume: 210.57 cm^3), the gamma pass rates were 77.07% for MC vs. AXB and 70.01% for MC vs. AAA. For patient 25 (PTV volume: 648.92 cm^3), the corresponding values were 70.50% and 69.97%, respectively. These results are consistent with our main findings that MC and AXB are in closer agreement than MC and AAA.

Because gamma analysis on patient CT dose is not a standard clinical practice and is less interpretable than DVH-based metrics, we have not included these exploratory results in the main text but are providing them here in response to the reviewer's request.

Comment 10:
What about providing graphs for stating distribution of the parameters such as CI, HI, GI, NTID?
Response 10:
Thank you for this helpful suggestion. We agree that graphical representation can improve the clarity of our results. In the revised manuscript, we have added Figure~\ref{fig:plan_quality_metrics}, which presents box-and-whisker plots of the Conformity Index (CI), Homogeneity Index (HI), Gradient Index (GI), and Normal Tissue Integral Dose (NTID) for the three algorithms (MC, AXB, and AAA).

These plots complement the numerical data in the text and tables by visualizing the distributions of these parameters and highlighting the differences among the algorithms. This addition improves the interpretability of our findings while maintaining consistency with the statistical results already presented.

Comment 11:
Please use the same color for the same organ in Figure 12.
Response 11:
Thank you for this comment. We understand the rationale for using the same color for the same organ; however, in this case, the dose–volume histogram (DVH) curves for the three algorithms (MC, AXB, and AAA) are very close to each other. If the same color were used for each organ, the curves would overlap and become visually indistinguishable.

To ensure clear differentiation between algorithms while maintaining organ identification, we retained distinct line styles and colors for each algorithm–organ pair. This approach has been widely used in previous DVH comparison studies and allows readers to distinguish both the organ and the algorithm simultaneously.

Reviewer #2:

Dosimetric comparison of VMAT lung cancer plans on Halcyon Accelerators using Monte Carlo, Acuros XB, and AAA algorithms

Manuscript Description

The submitted manuscript presents a valuable comparison of dose calculation algorithms—RayStation's Monte Carlo (MC) and Eclipse's Acuros XB (AXB) and AAA—in the context of Halcyon VMAT plans for lung cancer treatment. By recalculating treatment plans originally computed with Raystation's Monte Carlo algorithm using both AcurosXB and AAA, the authors directly compare these algorithms. Their work demonstrates good agreement between MC and AXB across multiple dosimetric metrics in clinical patient cases while AAA shows slight but clinically acceptable deviations. The study's strengths include the use of a real patient cohort in addition to a simple water phantom and rigorous statistical analysis. Overall, this manuscript makes a noteworthy contribution by demonstrating the reliability of algorithmic transitions for lung VMAT planning on the Halcyon platform.

However, the manuscript requires some major revisions before it can be considered for publication. I offer the following suggestions for revision:

General Comments

1. The introduction should provide a more compelling rationale for the research work. Especially, making it clear that this study is filling in the gap of the work Saini et al. (2021) would be important to show the originality of the study. Also, much of the discussion section about algorithm comparisons needs to be in the introduction, giving previous results as background knowledge.
2. The reasoning behind recalculating without dose renormalization for the prescription dose to cover 95% of the PTV does not seem persuasive enough. The method that "users at other institutions" use seem definitely more acceptable. Further argument backed up with references that support the claim that the CTV still receives the prescribed dose would be helpful for justifying the method used in the study.

Response to General Comments:

Response 1:

We thank the reviewer for this valuable comment. We agree that the relationship between our study and Saini et al. (2021) should be clarified to highlight our study's originality. Saini et al. (2021) primarily focused on the commissioning of RayStation for Halcyon using phantom-based measurements and did not include patient-based cross-TPS dose validation. In contrast, our work extends their findings by:

1. performing a large-scale **patient cohort study (n = 63)** with anatomically complex lung cancer cases,
2. incorporating a direct **cross-TPS comparison (RayStation MC vs. Eclipse AXB/AAA)**,
3. explicitly addressing the **clinical workflow of RayStation planning with Eclipse verification**, which reflects how Halcyon is used in many institutions.

We have revised the introduction to explicitly state these distinctions and moved part of the algorithm-comparison context from the discussion to the introduction to provide a clearer background.

Response 2:

We appreciate the reviewer's concern. In our institution, RayStation is the primary TPS used for treatment planning and clinical reporting across all linacs. Although Eclipse recalculation is mandatory for Halcyon delivery via the ARIA system, **dose renormalization is not performed** because it would alter the prescription dose parameters reported in RayStation, leading to discrepancies between the approved plan report and the delivered plan in ARIA. This practice is also consistent with the approach recommended in previous reports (e.g., Saini et al., 2021), where dose recalculation in Eclipse was performed **without renormalization** to preserve the integrity of the treatment plan.

Furthermore, Halcyon employs daily CBCT-guided setup correction, which ensures accurate CTV coverage even if small algorithm-related variations in PTV D95% are observed after recalculation in Eclipse. We have clarified this rationale in the revised Discussion section, with references added to support this workflow.

Specific Comments

Comment 1:
Halcyon prefixes: Choose either "haMC/haAXB/haAAA" or "MC/AXB/AAA," and define the "ha" prefix if retained.

Response 1:

Thank you for the comment. We have retained the "ha" prefix to indicate that these datasets correspond to Halcyon treatment plans. This distinction is useful because it differentiates these results from algorithm names (MC, AXB, AAA) themselves. We have clarified this definition in the Methods section to avoid any ambiguity.

Comment 2

CI vs PCI: Replace “CI” with “PCI” to accurately denote the Paddick Conformity Index to avoid confusion.

Response 2:

Thank you for pointing this out. We agree with the reviewer that “CI” should be replaced with “PCI” to explicitly denote the Paddick Conformity Index and avoid any potential ambiguity. We have revised the terminology throughout the manuscript, including in the Methods, Results, tables, and figure legends, to use “PCI” consistently.

Comment 3

Decimal formatting: In tables, ensure consistent decimal formatting (e.g., one decimal place).

Response 3:

Thank you for the comment. We have revised all tables in the manuscript to ensure consistent decimal formatting. All numerical values are now presented with one decimal place for clarity and uniformity.

Comment 4

Relative difference calculation: Express relative differences as (Comparing value − Reference) / Reference × 100%.

Consistent Reference: Choose an algorithm to serve as the reference (e.g., haMC) and apply it uniformly across all comparisons.

Response 4:

Thank you for these comments. We have revised the calculation of relative differences throughout the manuscript to use the following standardized formula:

$$\text{Relative Difference} = \frac{\text{Comparing Value} - \text{Reference}}{\text{Reference}} \times 100\%.$$

In addition, we have selected **haMC** as the consistent reference algorithm for all comparisons. The Methods section has been updated to clarify this, and all related tables, figures, and result descriptions have been modified accordingly. These changes ensure that the direction of the relative differences is uniform and that the results are presented in a clear and interpretable manner.

Comment 5

(p. 1, l. 4): Cite a source to support “VMAT has emerged as a key option...”

Response 5:

Thank you for this suggestion. We have added two references to support the statement that VMAT has emerged as a key option for the radiotherapy of NSCLC:

- Merrow et al. (2013) demonstrated that VMAT provides high-quality dose distributions for NSCLC with improved treatment efficiency compared to conventional techniques.

- Li et al. (2018) performed a dosimetric comparison between IMRT and VMAT in peripheral and central lung cancer and confirmed the superiority of VMAT in both target coverage and OAR sparing.

These references have been added to the Introduction.

Comment 6

(p. 2, l. 34): Add a reference when comparing AAA to Pencil Beam Convolution.

Response 6:

Thank you for the comment. In the revised manuscript, we have removed the mention of Pencil Beam Convolution (PBC) in this context.

Comment 8

(p. 4, l. 127): Include manufacturer name and location for the CT simulator.

Response 8:

Thank you for the comment. We have added the correct manufacturer name and location for the CT simulator in the revised manuscript.

Comment 9

Figure 2: Revisit PTV expansion for Patients 22, 55, and 61—small CTVs appear to have disproportionately large PTVs.

Response 9:

Thank you for the observation. We have reviewed the cases of Patients 22, 55, and 61. The apparently large PTV expansions are primarily due to the irregular shapes of the CTVs, which result in a larger relative margin effect during isotropic expansion (e.g., 5 mm) compared to more spherical targets. For example, the attached figure (not included in the manuscript) illustrates the

CTV and PTV for Patient 22, confirming that the margin application was geometrically consistent.

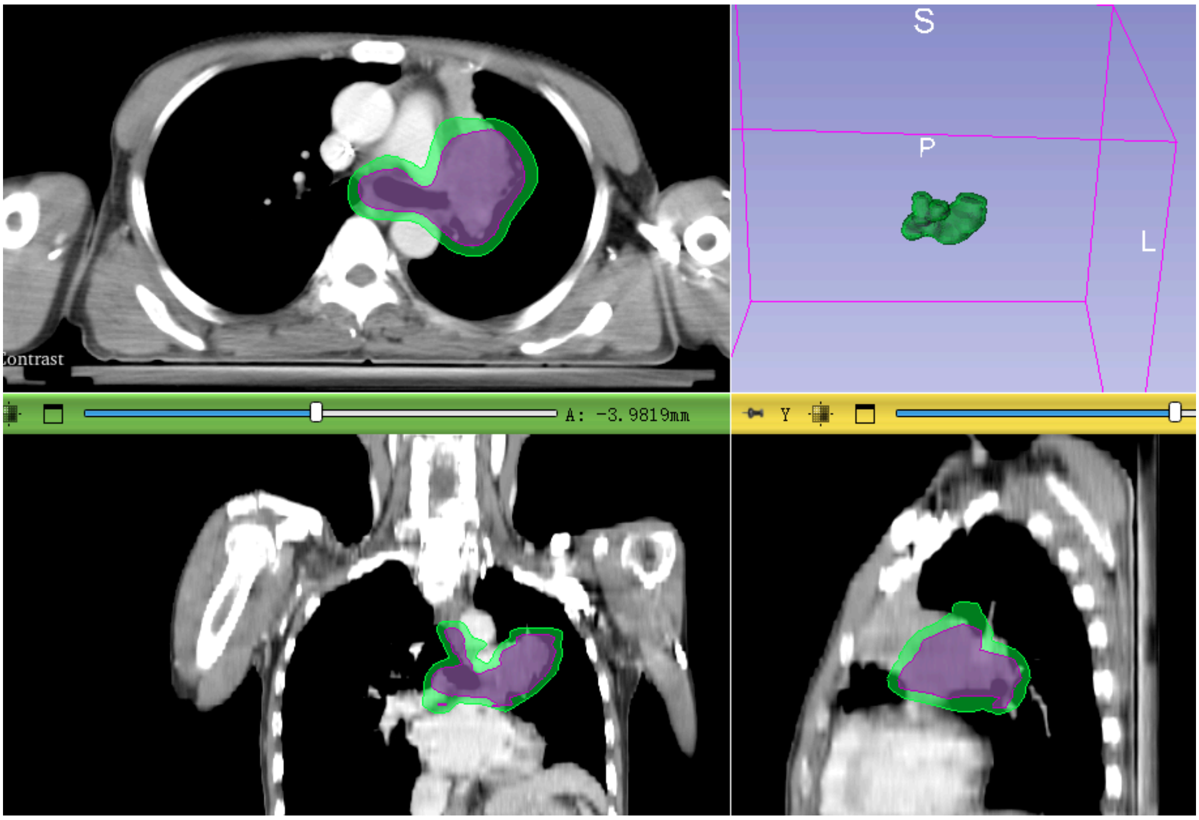

Comment 10

• (p. 5, l. 203): The mentioned NTID isn't shown in the OAR result.

• Table 3 typo: Correct "ClClinical" to "Clinical".

Response 11:

Thank you for the comment. We have clarified that NTID is reported alongside PCI, HI, and GI in the target plan quality metrics section rather than in the OAR results. To improve clarity, we have also added a new figure presenting NTID together with the other plan quality metrics based on the reviewer's previous suggestion.

Additionally, the typo in Table 3 ("ClClinical") has been corrected to "Clinical" in the revised manuscript.
