# Supplementary material for: Dosimetric comparison of Monte Carlo, Acuros XB, and anisotropic analytical algorithm for lung cancer plans on halcyon accelerators
Source: PLoS One. 2025 Oct 17;20(10):e0334329. doi: 10.1371/journal.pone.0334329 (PMC12533868; doi:10.1371/journal.pone.0334329)
Supplement: Attachment — Submitted filename: Response_to_reviewers_auresp_2.pdf [file pone.0334329.s006.pdf]

# Response to Reviewers

## Response to Reviewer #1

**Comment 1:**
I still think that the RayStation calculates based on the MC method but it doesn't represent the MC typically refers to in this research field. It would be more precise to describe the calculation as "RayStation MC" or an equivalent term rather than simply "MC".

**Response 1:**
We thank the reviewer for pointing out this important clarification. We agree that simply using the abbreviation "MC" could be misleading, as it might be interpreted as the generic, benchmark Monte Carlo algorithm rather than the specific clinical implementation in RayStation. To address this, we have revised the terminology throughout the manuscript. In the revised version, we now consistently describe this algorithm as "RayStation Monte Carlo (RMC)" at its first appearance (Abstract, Introduction, Methods), and use the abbreviation "RMC" in the subsequent sections, tables, and figures for clarity. This ensures precise differentiation between the RayStation implementation and the general Monte Carlo methodology commonly referred to in the research field.

---

**Comment 2:**
Please specify which accelerator model parameters were adjusted during the TPS commissioning.

**Response 2:**
We appreciate the reviewer's request for clarification. During TPS commissioning in RayStation, the accelerator model parameters were adjusted using the Physics module's automatic beam modeling function, which iteratively modifies the system parameters to best match the measured beam data. Specifically, the automatically tuned parameters included the photon energy spectrum, output factors (with correction for small and large field sizes), multi-leaf collimator (MLC) transmission, MLC tip width and MLC offset, and extra-focal/secondary source contributions. These adjustments ensured that the calculated percent depth dose (PDD) curves and lateral beam profiles matched those measured in a three-dimensional water phantom.

This modeling process was performed in collaboration with RaySearch support engineers and supervised by our senior physicist, with final acceptance testing conducted according to the AAPM Medical Physics Practice Guideline 5.a [Smilowitz et al., 2015]. The overall workflow closely followed the procedure described by Saini et al. (2021), who detailed the beam modeling process for Halcyon in RayStation. We have revised the Methods section to include these details in the manuscript.

---

**Comment 3:**
Authors previously responded to the comment on the rationale for using different ROI sizes in water phantom test but their clarification is not sufficient. They should provide the reasons why 3-cm-radius ROI is more appropriate to assess the high dose uniform region rather than 2 or 4 cm. Similarly, they should also clarify why 5-cm-radius ROI is suitable for representing the dose gradient or lower-dose regions than the ROIs with any other sizes.

**Response 3:**
We thank the reviewer for this constructive comment. Our selection of two spherical ROIs (3 cm and 5 cm in radius) was intended to provide representative volumes for algorithm comparison rather than to define "high-dose" versus "low-dose" regions. The 3-cm ROI was chosen to adequately represent the central uniform plateau of the 10×10 $cm^2$ field; a smaller ROI (e.g., 2 cm) would be too limited, while a larger ROI (e.g., 4 cm) would already extend toward the periphery. The 5-cm ROI was selected to extend into the peripheral region so that both the plateau and part of the dose gradient were included, which allowed us to examine algorithm performance across different parts of the beam profile. We have revised the manuscript to clarify this description and avoid the potentially misleading wording of "high-dose vs low-dose regions."

---

**Comment 4:**
It would be better to cite more relevant references for definitions or equations of HI and GI.

**Response 4:**
We thank the reviewer for this helpful suggestion. In the revised manuscript, we have added the appropriate reference for the definition of the homogeneity index (HI). Specifically, we now cite ICRU Report 83, which formally defines HI as ($D_2$% – $D_{98}$%) / $D_{50}$%. The gradient index (GI) remains referenced to Paddick (2006). This addition provides authoritative support for our methodological definitions.

---

**Comment 5:**
Please add reference for convincing that the AXB and AAA share similar dose calculation approximations, because they are implemented in the same TPS.

**Response 5:**
We appreciate the reviewer's helpful comment. We acknowledge that our previous explanation about the similarity between AXB and AAA was somewhat speculative and could be misleading. In the revised manuscript, we have removed this unnecessary interpretation and now report only the observed results, namely the high gamma pass rates between the two algorithms. This change keeps the description objective and focused on the experimental data.

---

**Comment 6:**
According to understanding, the RayStation MC shows better agreement with the AXB than with the AAA. However, some results (e.g. NTID, OAR doses) showed better agreement between the RayStation and AAA than between the RayStation and AXB. The authors should clarify this.

**Response 6:**
We thank the reviewer for this valuable observation. We agree that, while RayStation MC generally shows closer agreement with AXB than with AAA for target-related metrics (e.g., D95%, D98%, conformity index), some results such as NTID and certain OAR dose parameters appeared numerically closer to AAA. This phenomenon can be explained by the different sensitivities of these metrics: target-based indices mainly reflect high-dose regions (around the prescription dose of 60 Gy), where the particle transport modeling of AXB is more consistent with MC. By contrast, NTID and OAR-related doses are much lower (typically 10–20% of the prescription dose) and represent volumetric or peripheral dose contributions. These lower-dose metrics are more affected by smoothing and dose-spread approximations inherent in AAA, which in some cases can coincidentally yield values closer to MC.

Importantly, the magnitude of these differences remained small (usually within 1–2%), and given that the involved dose levels are far below the prescription dose, the clinical impact is negligible. The overall trend across the majority of dosimetric endpoints confirmed that AXB aligns more closely with RayStation MC. We have added clarification in the revised Discussion to reflect this point.

---

**Comment 7:**
Authors clarified that one of their strengths over previous researches were that the validation is specified for Halcyon accelerator. I assume there are not substantial differences in beams of the same energy, such as 6 MV FFF. The differences between the accelerator types, C-type or O-type, are not expected to affect the TPS dose calculation accuracy. Could the authors clarify whether significant differences exist?

**Response 7:**
We thank the reviewer for raising this important point. We agree that, from a fundamental physics perspective, 6 MV FFF beams generated by Halcyon and by conventional C-arm linacs should not exhibit substantial differences in terms of intrinsic beam quality. The main distinction lies in the modeling of the dual-layer MLC and the closed design of the Halcyon platform.

The motivation for our study was therefore not based on an assumption of different beam physics, but rather on the specific clinical workflow constraints of Halcyon. In our institution, plans generated in RayStation must be recalculated in Eclipse before approval and delivery in the ARIA record-and-verify system. This step is not required for C-arm accelerators, where Eclipse plans can be directly approved and delivered. As a result, Halcyon introduces a unique cross-TPS recalculation process that warrants careful validation.

To our knowledge, large-cohort validations of RayStation-to-Eclipse workflows for Halcyon have not been reported previously. Thus, our work provides practical confirmation that algorithmic consistency is preserved in this dual-TPS setting, supporting safe and effective clinical implementation for Halcyon-based treatments.

We have also revised the Introduction to emphasize this workflow-driven motivation rather than beam differences.

---

**Comment 8:**
Authors suggested one of the advantages of RayStation over Eclipse is integrated workflow. But the Eclipse seems to be more integrated in the case of medical facilities equipped exclusively with Varian accelerators.

**Response 8:**
We appreciate the reviewer's comment and agree that Eclipse provides a more integrated workflow for medical facilities that operate exclusively with Varian accelerators, particularly for Halcyon. In our institution, however, the situation is somewhat different. Historically, our department transitioned from Pinnacle to RayStation, and RayStation has since become the primary platform for treatment planning and evaluation across multiple accelerators, including Trilogy (Varian), TrueBeam, and Infinity (Elekta). As a result, our clinical staff (physicians, physicists, and dosimetrists) are highly experienced and efficient with RayStation, and it remains the routine TPS for most patients.

Although Eclipse is also available, the number of purchased licenses is limited, and it is mainly used for Halcyon delivery approval. Given the large patient load and our well-established RayStation workflows, a substantial proportion of Halcyon plans are still designed and optimized in RayStation before being recalculated in Eclipse for final approval. Our study therefore reflects this real-world practice in our center rather than a general claim of superiority of RayStation over Eclipse.

We have revised the Introduction and Discussion to clarify this workflow-driven motivation and to avoid the impression of claiming superiority of RayStation over Eclipse.
